# Ankyrin‐2 genetic variants: A case of Ankyrin‐B syndrome

**DOI:** 10.1111/anec.12933

**Published:** 2022-02-28

**Authors:** Jin Song, Bryan Richard Sasmita, GuoLan Deng

**Affiliations:** ^1^ 117972 Department of Cardiology The First Affiliated Hospital of Chongqing Medical University Chongqing China

**Keywords:** ankyrin 2, Ankyrin‐B syndrome, genetic mutations, inherited cardiac arrhythmias

## Abstract

Inherited cardiac arrhythmias (ICA) have become one of the leading causes of sudden cardiac death in people under 40 years old. Variants in the ankyrin‐B or ankyrin‐2 genes will result in several cardiac arrhythmias ranging from sinus node dysfunction to life‐threatening arrhythmias. In this case study, we report a typical ankyrin‐2 variant, in which ventricular tachyarrhythmias might be reproduced through exercise or stress tests.

## 1 CASE REPORT

A 20‐year‐old Asian man, due to “exercise‐related syncope for 4 years” was presented to the outpatient department. He was having syncopal episodes with spontaneous resolution in a few minutes of rest. Before the syncope, he may experience chest discomfort, fatigue, and diaphoresis. He was a non‐smoker, and there was no history of drug abuse. Family history was negative for sudden cardiac death (SCD) or cardiac diseases.

**FIGURE 1:**
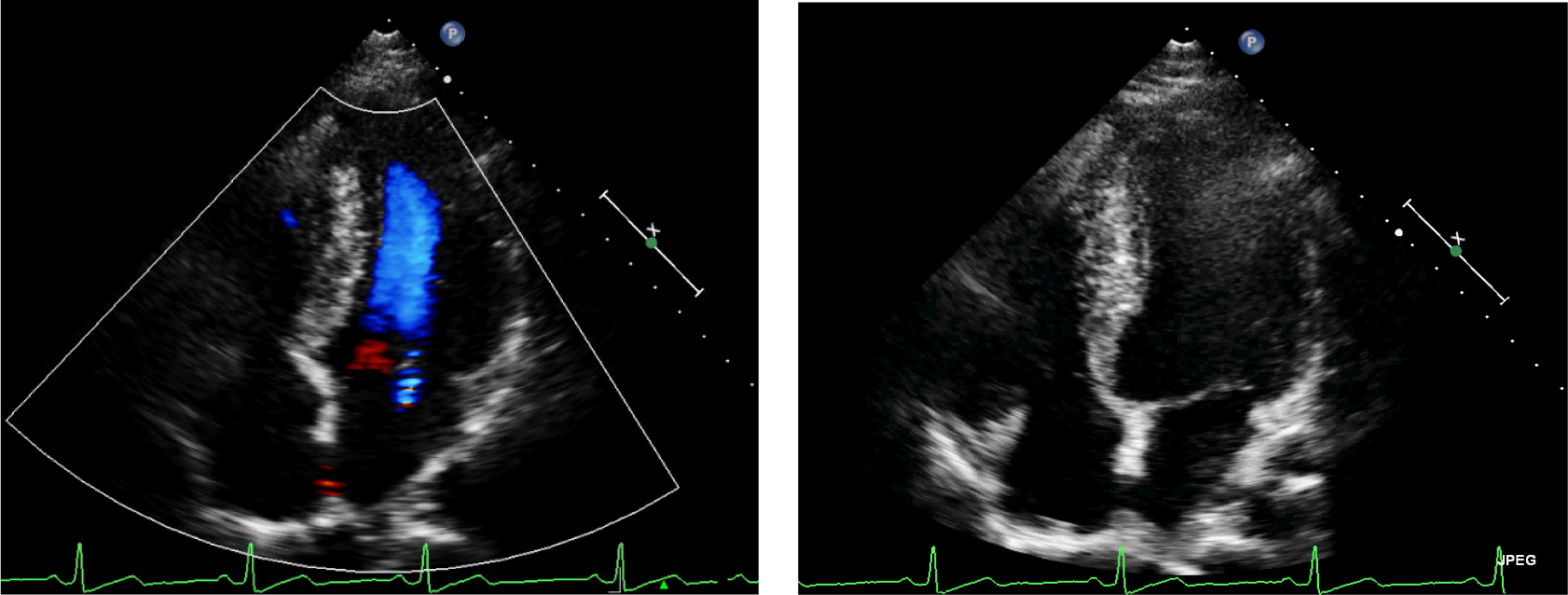
Cardiac Echocardiography showed asymmetric left ventricular hypertrophy with diastolic dysfunction

**FIGURE 2:**
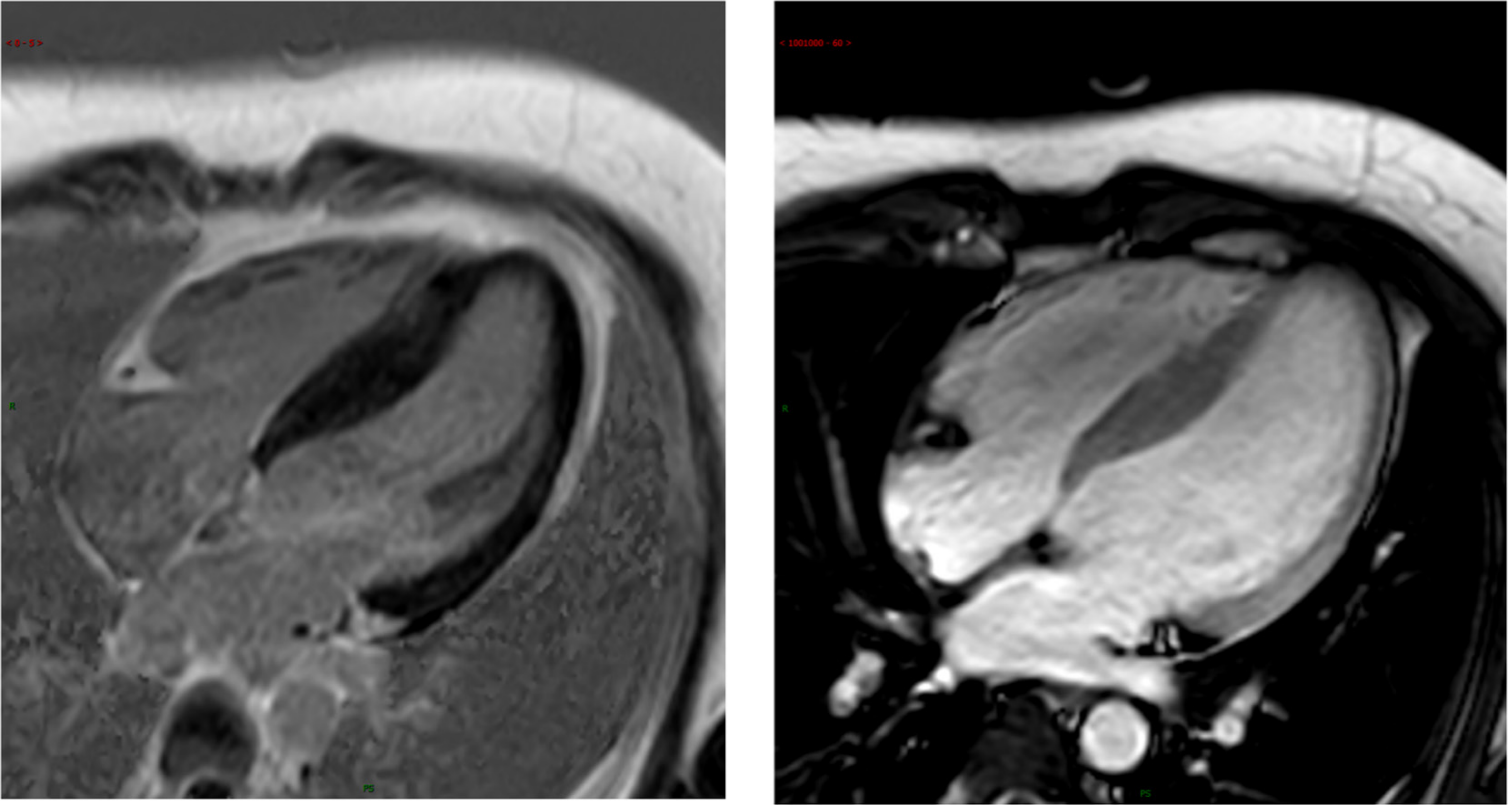
Cardiac MRI displayed normal structure and function, with a 17‐mm interventricular septum

The physical examination was presented without any abnormal findings. Admission laboratory findings include thyroid function, and cardiac biomarker was normal. Resting electrocardiogram (ECG) showed a resting heart rate (HR) of 78 beats/minute (bpm). Cardiac Echocardiographic and MRI showed asymmetric left ventricular hypertrophy, decreased diastolic function, normal cardiac structure, therefore, non‐obstructive hypertrophic cardiomyopathy was diagnosed (Figures 1 and 2). Coronary angiography and pulmonary angiography examinations were done to rule out the possibility of coronary artery disease or pulmonary embolism, the results were negative.

**FIGURE 3:**
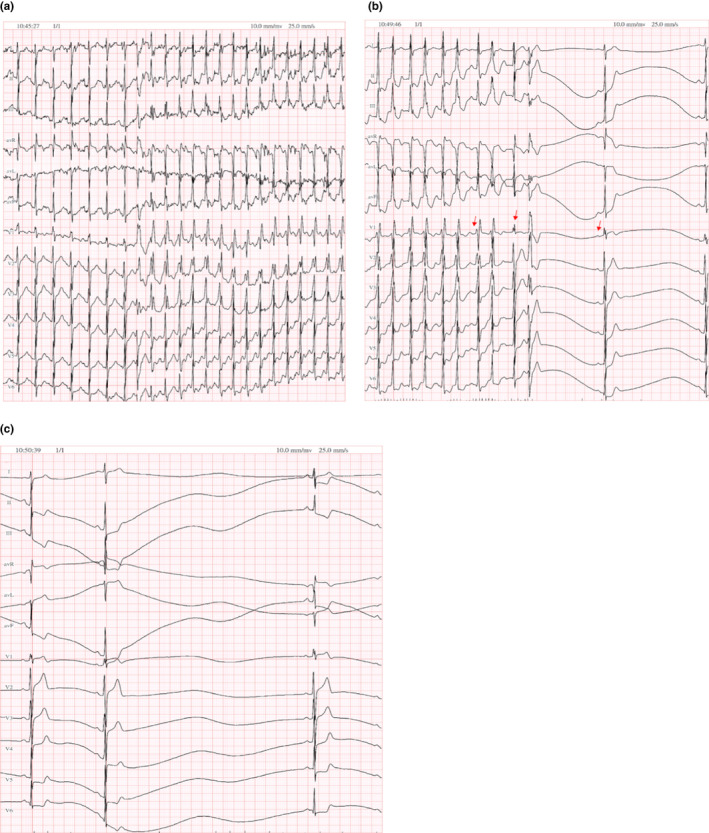
(a) During the second treadmill stress test, HR rise up to 160 bpm, and then ventricular premature beat triggered a wide‐QRS complex tachycardia (ventricular rate 218 bpm); (b) Termination of ventricular tachycardia followed with sinus arrest (red arrow: sinus rhythm 83 bpm); (c) Termination of ventricular tachycardia followed with cardiac arrest, junctional escape beat, R–R interval greater than 4 s, and binodal disease

As we suspect, the possibility of cardiac abnormal rhythm, under the supervision of a cardiologist, as well as advanced life support equipment, we ordered a treadmill stress test. The examination was done with Bruce's protocol stress test, during examination, patient's heart rate (HR) was stimulated up to 160 bpm, and ECG showed wide‐QRS complex tachycardia with ventricular rhythm 218 bpm with obvious A–V dissociation. The stress test was immediately terminated, and 4 min after patient's ECG showed a sequence of changes from sinus arrest to cardiac arrest, and eventually to junctional escape rhythm; therefore, we consider the patient had a binodal disease (BND), which coexistence of atrioventricular conduction disturbances with sick sinus syndrome (SSS) (Figure 3a–c). After a thorough examination, our initial diagnoses were 1. Non‐obstructive hypertrophic cardiomyopathy; 2. Catecholaminergic ventricular tachycardia.

**FIGURE 4:**
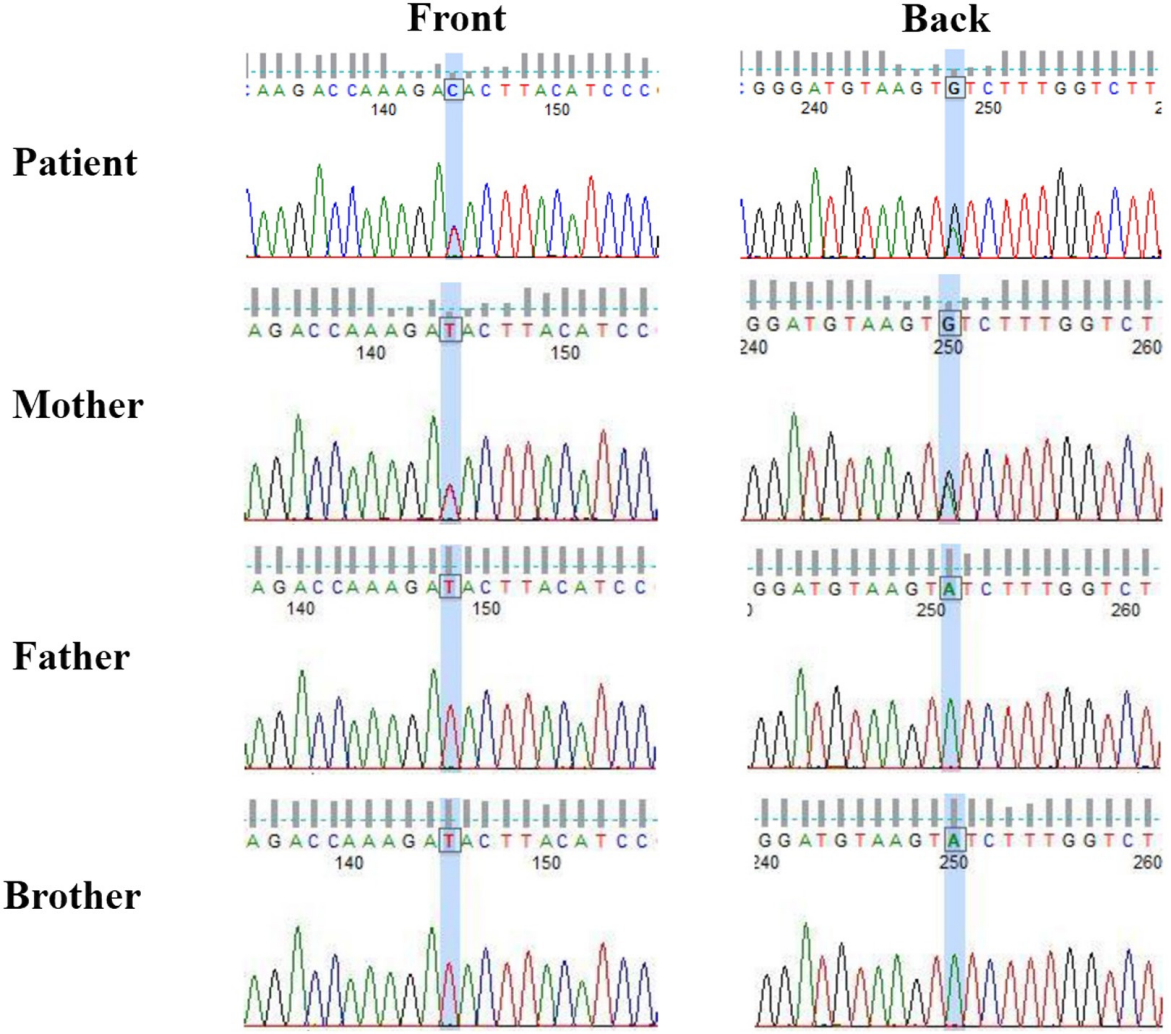
Genetic examination showed that the patient had ankyrin 2 (ANK2) c.10310T > C variant, while patient's mother presented with ANK2 compound heterozygous mutation

As the patient's cardiac echocardiography was remarkable, genetic examination was performed to rule out inherited cardiac arrhythmias. Genetic examination was carried out on patient's family and himself. Around 5ml of blood was collected in K2‐EDTA tubes, following accepted principles for blood drawing and blood collection. Genetic sequencing was performed on the Illumina HiSeq system, while genomic analysis was performed by using WuXi NextCode Clinical Sequence Analyzer (CSA) to identify clinically relevant phenotype‐associated genes and variants from family members. After DNA capture, sequencing was done by using Illumina Cluster and SBS reagents to reach a mean coverage depth of more than 90× per sample, with 95% of the targeted regions covered at least 20 times. Routine exome sequencing was performed under The American College of Medical Genetics and Genomics (ACMG) “Interpretation of Sequence Variants Standards and Guidelines” and Clinical Laboratory Improvement Amendments (CLIA ID: 99D2064856). Human Genome Variation Society (HGVS) nomenclature was used to describe genetic sequence variants. Our genetic examination showed that the patient had ankyrin 2 (ANK2) c.10310T > C variant, while patient's mother presented with ANK2 compound heterozygous mutation. Negative findings were demonstrated by other family members (Figure 4).

After diagnosis was confirmed, the patient was given metoprolol succinate 23.75 mg QD, and we advised the patient to do a regular check‐up every 1–3 months. Unfortunately, the patient was not doing a monthly check‐up at our hospital; therefore, we do a follow‐up to the patient, and he mentioned that he deliberately stopped the medication, moreover, the aforementioned symptoms still persist, especially after strenuous exercise.

## 2 DISCUSSION

In this case report, we presented a patient with what we called “Ankyrin‐B syndrome”, a type of inherited cardiac arrhythmias (ICA) that account for 1% of the cases. ICA are abnormal heart rhythms that are passed down from family members, ranging from benign to life‐threatening arrhythmia. Most people with ICA are young people and may present with or without structural changes of the heart. ICA has long been associated with abnormality in the cardiac ion channels and sodium channel integral membrane protein, which disrupt normal cardiac conduction system, hence arrhythmias were manifested. However, this theory was not last long until Schott in 2003 and Mohler in 2004 found persons with a mutation in the ankyrin‐B were presented with the long‐QT syndrome (LQTS) during ECG examination (Mohler et al., 2004).

Ankyrin‐B syndrome (ABS) initially was found in patients with a variant in ankyrin‐B E1425G, which results in type 4 form of LQTS, therefore, at first Ankyrin‐B syndrome was also known as Type 4 LQTS (Mohler et al., 2004). However, in recent years with more advanced technology, as well as a higher incidence of unexplained cardiac arrhythmias in young people, an increasing attention has been directed toward the possibility of other genetic mutations producing life‐threatening arrhythmia. In the recent study conducted by Mohler, et al (Mohler et al., 2007), they found out Ankyrin‐B variants not only presented with long‐QT syndrome, but may also manifest as bradycardia, conduction block, atrial fibrillation, and catecholaminergic polymorphic ventricular tachycardia (Cunha et al., 2007). Mohler and colleagues found out that Ankyrin‐B loss‐of‐function variants may be classified according to its clinical phenotypes (Cunha et al., 2007). For example, ankyrin‐B variants of T14041 were presented with bradycardia, atrial fibrillation (AF), drug‐induced TDP, and LQTS, while variants of V1516D were associated with AF, bradycardia, BS, drug‐induced LQTS, and exercise‐induced VT. Unfortunately, there are several genetical variants of ankyrin‐B, which is still unknown of its clinical phenotype, for example, a mutation in G1406C, R1450W, L1503V, T1552N, V1777M, and E1813K (Mohler et al., 2007).

Complex mechanisms involved in how ankyrin‐B mutations result in several life‐threatening arrhythmias, with a recent study has demonstrated that dysfunction of ankyrin‐B may result in abnormal sinoatrial node (SAN) electrical activity, thereby causing bradycardia (Mohler et al., 2005). As for ventricular tachyarrhythmia, several studies believe 2 main mechanisms come to exist. First, ankyrin‐B mutations presented with the phenotypes of cardiac voltage‐gated sodium channel (SCN5A), a variant that abolishes binding of Na_v_1.5 with ankyrin‐G resulting in Brugada syndrome. Second, loss‐of‐function ankyrin‐B presented with decreased expression of Na/Ca exchanger, which led to sarcoplasmic reticulum calcium overload, and eventually increased catecholamine‐induced afterdepolarizations (Cunha et al., 2007; Mohler et al., 2005; Mohler et al., 2003).

Indeed, diagnosis of ABS has been challenging; therefore, we suggested arrhythmic patients under 40 years old with normal cardiac structure and function to undergo genetic testing for early screening and prevention of possible ICA. The variability of abnormal electrical conduction has made it difficult to find the optimal medical management for ABS. For example, in our case study, as the patient during stress test mainly presented with LQTS; therefore, we advised the patient to avoid strenuous exercise, and we gave beta‐blocker as the mainstay treatment. However, problems arise if ABS presents as sinus bradycardia, which in this case if we use a beta‐blocker will only make the condition worsened. Insertion of a permanent pacemaker may be indicated in individuals with sinus node dysfunction, while implantable cardioverter‐defibrillator (ICD) should be considered for individuals with life‐threatening arrhythmia that can't be controlled with anti‐arrhythmic agents. ABS due to their complexity in nature, as well as difficulty in diagnosis and treatment, have become one of the leading causes of sudden cardiac death in people under 40 years old, therefore, early screening, diagnosis, prevention, and treatment for every ABS individual should be done to reduce the risk of sudden cardiac death.

## CONFLICT OF INTEREST

The authors declare no competing interests.

## AUTHOR CONTRIBUTIONS

J.S and S.B.R contributed to the conception and design of the work and drafted the manuscript. G.L.D revised and directed the manuscript.

## ETHICAL APPROVAL

This study was performed in accordance with the principle of the Declaration of Helsinki and the research protocol was approved by the institutional ethical review board of the first affiliated hospital of Chongqing medical university (April 29, 2020, No.2020‐233).

## INFORMED CONSENT

A written informed consent for publication was obtained from the patient.

## Data Availability

All data relevant to the study are included in the article or uploaded as supplementary files. Data can also be requested from the corresponding author.
